# Results from COG0201: A Randomized, Double‐Blind, Placebo‐Controlled, Parallel‐ Group, Phase 2 Study to Evaluate the Safety and Efficacy of CT1812 in Subjects with Mild to Moderate Alzheimer’s Disease

**DOI:** 10.1002/alz.089155

**Published:** 2025-01-09

**Authors:** Everard G.B. Vijverberg, Susan Catalano, Mary E. Hamby, Michael Grundman, Jennifer Iaci, Theresa Devins, Anthony O Caggiano

**Affiliations:** ^1^ Brain Research Center, Amsterdam Netherlands; ^2^ Alzheimer Center Amsterdam, Neurology, Vrije Universiteit Amsterdam, Amsterdam UMC location VUmc, Amsterdam, North Holland Netherlands; ^3^ Capsida Therapeutics, Thousand Oaks, CA USA; ^4^ Cognition Therapeutics, Inc, Pittsburgh, PA USA; ^5^ Global R&D Partners, LLC, La Jolla, CA USA; ^6^ Cognition Therapeutics, Purchase, NY USA

## Abstract

**Background:**

CT1812 is an experimental therapeutic sigma‐2 receptor modulator in development for Alzheimer’s disease (AD) and dementia with Lewy bodies. CT1812 reduces the affinity of Aβ oligomers to bind to neurons and exert synaptotoxic effects. This phase 2, multi‐center, international, randomized, double‐blind, placebo‐controlled trial assessed safety, tolerability and effects of CT1812 on cognitive function in individuals with AD.

**Method:**

Consenting participants with confirmed brain amyloid and baseline MMSE scores of 18 to 26 were randomized equally to receive oral placebo, 100 or 300mg of CT1812 daily for 6 months. Exploratory efficacy outcomes included ADAS‐Cog11 and 13, Neurophysiological Test Battery, ADCS‐Clinical Global Impression of Change and ADCS‐Activities of Daily Living. The primary statistical analysis is treated (combined 100 and 300mg CT1812) versus placebo change from baseline in ADAS‐Cog11. Canonical AD biomarkers were assessed in CSF and plasma.

**Result:**

One hundred fifty‐three participants were randomized. An interim analysis of the first 24 participants was performed where baseline demographics included mean age of 71.7 years, 15 females and a mean MMSE of 21.1. In the interim analysis there were twenty‐two TEAEs (7, 8, 7 in the 100, 300mg CT1812 and placebo groups, respectively) reported and in the CT1812 groups, all TEAEs were mild or moderate and there were no treatment related SAEs. There was a trend within these 24 participants for slower progression at six months in the CT1812 treated group with ADAS‐Cog11 improved 3.1 points relative to placebo according to the prespecified statistical model. Complete data from all participants including AEs, cognitive performance and canonical biomarkers will be presented with the ADAS‐Cog11 change from baseline relative to placebo for the pooled CT1812 treated group prespecified as the first cognitive assessment with ApoE4 status as a covariate.

**Conclusion:**

CT1812 is an oral sigma‐2 modulator that displaces Aβ oligomers from neurons. Encouraging trends have been observed in previous studies (NCT03493282, NCT03522129, NCT04735536) and in an interim analysis of this phase 2 trial (NCT03507790). This trial will provide proof of concept data indicating if CT1812 can slow progression in people with mild to moderate AD. This study was supported by a grant from the NIH (AG058660).

